# Examining HPV vaccination behavior among young adults: Insights from applying the Health Belief Model

**DOI:** 10.1371/journal.pone.0312700

**Published:** 2024-11-01

**Authors:** Oluwafemifola Oyedeji, Kristina W. Kintziger, Cary M. Springer, Samantha Ehrlich, Jill Maples, Justin Gatwood, Cristina S. Barroso

**Affiliations:** 1 Department of Public Health, The University of Tennessee, Knoxville, Tennessee, United States of America; 2 Department of Environmental, Agricultural, and Occupational Health, College of Public Health, University of Nebraska Medical Center, Omaha, Nebraska, United States of America; 3 Research Computing Support, Office of Innovative Technologies, The University of Tennessee, Knoxville, Tennessee, United States of America; 4 Department of Kinesiology, Recreation, and Sport Studies, The University of Tennessee, Knoxville, Tennessee, United States of America; 5 Department of Obstetrics and Gynecology, The University of Tennessee Graduate School of Medicine, Knoxville, Tennessee, United States of America; 6 US Health Outcomes GlaxoSmithKline, Philadelphia, Pennsylvania, United States of America; 7 Department of Health, Behavior and Society, University of Texas School of Public Health San Antonio, San Antonio, Texas, United States of America; 8 College of Nursing, The University of Tennessee, Knoxville, Tennessee, United States of America; University of Udine: Universita degli Studi di Udine, ITALY

## Abstract

**Background:**

Despite evidence-based recommendations for HPV vaccination, uptake among young adults is suboptimal. Limited research has explored factors that may influence HPV vaccination among young adults, as well as associated promotion and informational preferences in this group. This study aimed to examine factors associated with HPV vaccination among young adults and preferences for HPV vaccine information and promotion.

**Methodology:**

This study utilized a cross-sectional design to examine factors related to HPV vaccination among young adults on a university campus in Tennessee. Data were collected via an online survey administered to students, aged 18–26 years, from March–May 2023. Study measures included sociodemographic characteristics, health/healthcare-related factors, and perception-related measures. Data analysis included descriptive statistics and multivariable logistic regression analysis.

**Results:**

Out of 402 participants that completed the survey, 209 reported being vaccinated against HPV. In the adjusted model, variables associated with HPV vaccination were perceived risk (adjusted OR [aOR] = 1.12; 95% confidence interval [CI]:1.05, 1.20), perceived barriers (aOR = 0.73; 95% CI: 0.66, 0.81), higher HPV knowledge (aOR = 1.17; 95% CI: 1.03, 1.33), and receipt of healthcare provider recommendations (aOR = 12.90; 95% CI: 7.08, 23.51).

**Conclusions:**

Study findings suggest that those who are vaccinated were more likely to report receiving healthcare provider recommendations, low perceived barriers, higher HPV knowledge and perceived risk. Future HPV vaccination promotion efforts for young adults may consider increasing HPV vaccine knowledge and awareness, addressing barriers, and strengthening provider recommendations.

## Introduction

Human papillomavirus (HPV) vaccination is recommended for the prevention of cancers associated with persistent HPV infection including cervical, oropharynx, vulvar, vaginal, anal, and penile cancers [1, 2]. According to the Advisory Committee on Immunization Practices (ACIP), HPV vaccination is routinely recommended for children as early as 9 years through age 26 years [3]. Despite evidence-based recommendations, HPV vaccination rates are below desirable levels based on the 80% target set by Healthy People 2030 for adolescents [4]. In 2022, about 76% of adolescents aged 13–17 years initiated and 63% completed the HPV vaccine series [5]. For adults aged 19–26 years, about 53% of females and 26% of males received at least one dose of the HPV vaccine in 2018 [6]. Specifically for college students, data from the American College Health Association’s National College Health Assessment (Fall 2019 to Spring 2023) showed that 53.6% reported HPV vaccine completion, 18.7% reported not starting the series, 4.5% initiated the series, and 23.3% did not know their vaccination status [7].

Improving HPV vaccination rates is critical given that over 47,000 HPV-associated cancers are newly diagnosed each year in the United States (U.S.) [8]. HPV vaccination could prevent 90% of HPV-related cancers, including those without recommended cancer screening tests [9]. University campuses provide a distinctive opportunity to enhance the health of young adults through disease prevention and health promotion since college students constitute an integral part of the U.S. population and often encounter various health challenges [10]. Additionally, students frequently have high access to healthcare due to campus health services or subsidized student health insurance plans and can make health decisions independently [11]. Young adults having the option to decide on their health-related choices is important given that previous research has shown that parental or caregiver perceptions and attitudes regarding the HPV vaccine may influence uptake during childhood [12]. In its Healthy Campus 2020 initiative, the American College Health Association included increasing HPV vaccination coverage as part of national student objectives for improving health on campuses, underscoring the importance of vaccination promotion efforts among young adults [13].

Previous studies conducted among young adults have shown that individual attitudes and perceptions play a significant role in the decision to receive the HPV vaccine. For example, individuals who believe that the HPV vaccine is beneficial and can prevent high-risk HPV infections may be more likely to receive it [14]. Conversely, individuals who have a low perception of the risk of HPV infection or who believe that HPV is not severe may be less likely to receive the vaccine [14, 15]. Additionally, studies have shown that inadequate knowledge and awareness about HPV are common reasons for not receiving the vaccine [16], and recommendations from healthcare providers are often influential in the decision-making process [17]. To further examine individual-level determinants of HPV vaccine uptake, including perceptions and beliefs, this study applied the Health Belief Model (HBM). The HBM posits that perceived severity, perceived risk, perceived barriers, perceived benefits, cues to action, and self-efficacy are associated with the adoption of health behaviors [18]. In addition, the HBM framework also acknowledges the role of modifying factors related to health behaviors, such as age, race or ethnicity, socioeconomic status, and knowledge [18]. The application of the HBM framework to explain young adults’ behaviors regarding HPV vaccination allows the capturing of critical individual-level factors, including beliefs or perceptions that have been shown to influence behavioral change and intention [18].

In the United States, geographical differences exist in cancers associated with HPV and vaccination coverage [19, 20]. Data reports from the U.S. Cancer Statistics Working Group indicate disproportionately higher HPV-associated cancer incidence rates and lower HPV vaccination coverage in several southern states [19]. Specifically for Tennessee, the incidence rate of cancers associated with HPV was 14.4 per 100,000 persons from 2016 to 2020 [19]. This rate is higher than the national average and identifies Tennessee as one of the states with the highest incidence rate of HPV-associated cancer, ranking ninth highest in the country [19]. Likewise, HPV vaccination coverage in Tennessee is below the desired target as only about 64% of adolescents aged 13–17 years have been fully vaccinated against HPV in 2022 [20]. Previous HPV vaccine research among young adults has been conducted in Southern U.S. or Appalachian regions [21–23]. However, fewer studies have explored contextual factors influencing HPV vaccine uptake among adult residents in Tennessee [24, 25]. Other studies that included study population in Tennessee have focused on parents or caregivers [26, 27]. Examining the perspectives of young adults in different regions provides opportunities to tailor vaccination interventions or strategies by considering unique community contexts. Previous research conducted in Appalachia region across three states, namely Kentucky, Ohio, and Pennsylvania, revealed nonuniform cancer-related perceptions and beliefs among these three regions [28]. This highlights the significance of understanding region-specific factors that may influence health-related beliefs or behaviors, such as vaccine uptake. To improve vaccination rates and ultimately reduce HPV-associated cancer, it is essential to explore state or region specific contextual factors that may influence HPV vaccination. Additionally, limited research has been conducted to describe preferences for HPV vaccine information and promotion strategies among young adults. Therefore, this study aims to describe factors influencing HPV vaccine uptake and preferences for HPV vaccine information and promotion strategies among young adults on a university campus in Tennessee.

## Methods

### Study design

We utilized a cross-sectional design to examine factors related to HPV vaccination among young adults at a university in Tennessee. An online survey was developed to determine predictors of HPV vaccination among young adults. Eligible participants for this study included enrolled undergraduate and graduate students between 18–26 years old.

### Survey development and data collection

We drafted survey items based on the constructs of the HBM framework, previous studies [29–31], and qualitative research findings on HPV vaccination experiences among young adults. The draft survey items were reviewed for content and contextual relevance by experts familiar with HPV vaccination research among young adults and revised based on feedback. Thereafter, survey items were tested using cognitive interviewing techniques among a sample of students similar to the study population (i.e., students between 18–26 years). Cognitive interview is a method used to qualitatively evaluate survey items by understanding how participants respond to questions with the goal of improving survey items [32, 33].

Students were invited for the cognitive interview by sending email invitations through the author’s departmental mailing list. A total of six students expressed interest and consented to participate in the cognitive interview. The cognitive interview was conducted via zoom with transcripts automatically generated. The survey items were shared with the participants via Zoom. During the interviews, “think aloud” and “verbal probing” techniques was used to elicit responses. The “think aloud” technique involved asking participants to express the thoughts and ideas that arise as they respond to a question [32, 33]. In other words, participants discussed the process used to arrive at an answer to a question [32, 33]. The verbal probing technique involved asking participants about their interpretation of the survey items, the definition of keywords/phrases in a survey item and paraphrasing a survey item in their own words [32, 33]. Findings from the cognitive interviews were used to revise and develop the final survey items. Final survey items are included as S1 File.

We collected data using an online survey administered through Qualtrics (Provo, UT). Study participants were recruited using a convenience sample of 4000 email addresses of enrolled students aged 18–26 years obtained from the university for research purposes. An invitation to participate in the study and the link to the survey were sent to the email addresses. The online survey took approximately 10–15 minutes, and participants received a $10 gift card for study participation. Survey was administered from March 23, 2023 to May 4, 2023.

**Ethical considerations.** This study was approved by the University of Tennessee Knoxville Institutional Review Board (UTK IRB-22-07245-XM). Participants completed a written informed consent before starting the survey in Qualtrics. An IRB approved informed consent statement was placed at the beginning of the survey with the option for respondents to participate in the study. Participants who selected the option "I agree to participate" after reading the IRB approved consent statement proceeded to complete the survey. Participants who selected "I do not agree to participate" were exited out of the survey.

### Study measures and variables

The dependent variable for this study is HPV vaccination status as reported by participants. Survey respondents were asked if they had received the HPV vaccine, which was recoded into two categories with responses “yes” as vaccinated and “no” and “do not know” as unvaccinated. Variables were recoded due to small sample sizes for some categories. Potential predictors were grouped into sociodemographic characteristics, health/healthcare-related factors, and perception-related measures. Sociodemographic characteristics included age, sex assigned at birth (male/female), relationship status, race, and ethnicity. Relationship status was recoded into two categories as single or partnered (dating/married/living with a partner). Race was recoded into two categories as White and Other (Black, Asian, and more than one category). Health/healthcare-related factors included having a regular place to receive health care (yes/no), receipt of healthcare provider recommendations (yes/no), and HPV knowledge. HPV knowledge was assessed by a set of seven questions adapted from previous literature [30, 34], and a knowledge score was computed with a higher score indicating higher knowledge.

For perception-related measures, participants were asked to rate their level of agreement using five-point Likert scale answer choices ranging from strongly agree (5) to strongly disagree (1). Perception-related measures were HBM constructs, including perceived risk (e.g. “I consider myself at risk for HPV infection”), perceived severity (e.g. “Being infected with HPV would be very serious for me”), perceived benefit (e.g. “I think the HPV vaccine will be beneficial to me”), perceived barrier (e.g. “How much would the following factors prevent you from getting vaccinated for HPV; concerns about the possible side effects of the HPV vaccine”), and self-efficacy (e.g. “I feel confident in my ability to get vaccinated for HPV”) [29–31]. A composite score was computed by adding answer choices for the items with higher scores indicating higher perceptions. Summary statistics and internal reliability were examined for the scales (see supporting information). Additionally, participants were asked to rate their level of agreement regarding different HPV vaccination promotion strategies and select various preferred options for HPV vaccine information sources and format (non-mutually exclusive responses).

### Statistical analysis

We conducted descriptive statistics, which involved computing medians and standard deviations for continuous variables and frequencies and percentages for categorical variables. We used chi-squared tests to examine differences in participants’ characteristics by vaccination status. Potential correlation between variables was examined using Spearman rank correlation analyses (no significant strong correlations were detected between the variables, with all *r* values ≤ 0.5*)*.Respondents with missing values for variables of interest were excluded from final analysis. Multivariable logistic regression modeling was conducted to examine which predictor variables were associated with HPV vaccination status. The initial model building process involved bivariate associations between each independent variable and HPV vaccination status along with calculation of unadjusted odds ratios (OR) and 95% confidence intervals (CI). The threshold for variables to remain in the full model was set at p-value ≤ 0.05. Manual backward elimination procedure was used for fitting the final multivariable logistic regression model. This process involved removal of non-significant variables starting with highest p-value until all variables in the model were significant at a p-value ≤ 0.05. Regression coefficient change was observed as variables are removed from the model. Potential confounders were retained in the model if removal resulted in ≥ 20% change in other variables in the models even when the p-value was not significant. Adjusted odds ratios (aOR) and 95% confidence intervals were computed from the final model. The Hosmer-Lemeshow test was used to assess the goodness of fit for the final model [35]. Data analysis was done using SPSS (Version 26.0, IBM Corp, Armonk, NY, USA) and STATA (Version 16, StataCorp LLC, College Station, TX).

## Results

### Characteristics of participants

Table 1 presents the characteristics of study respondents (N = 402). The median age of participants was 20 ± 2 years, and most participants were female, White, non-Hispanic, and partnered. About 61.8% of study participants currently have a regular place of care and 94.5% currently have health insurance. The knowledge score ranged from zero to seven with a mean of 3.8. Most participants reported receipt of HPV vaccine recommendations from their healthcare provider (62.2%). About half of participants (51.5%) reported receiving the HPV vaccine, 20.1% reported that they had not received the HPV vaccine, and 27.9% were unsure about their vaccination status. A higher proportion of females reported being vaccinated compared with males.

**Table 1:** Characteristics of participants of a university survey, Tennessee (N = 402).

	Total		Vaccinated		Unvaccinated		Chi-square Test
	N*	%	n	%	n	%	p-value
**Sex**							
Female	301	74.9	174	57.8	127	42.2	<0.001
Male	101	25.1	35	34.7	66	65.3	
**Race**							
White	352	89.1	188	53.4	164	46.6	0.253
Others ^a^	43	10.9	24	55.8	19	44.2	
**Ethnicity**							
Hispanic	24	6.1	14	58.3	10	41.7	0.675
Non-Hispanic	370	93.9	194	52.4	176	47.6	
**Relationship Status**							
Single	188	46.8	89	47.3	99	52.7	0.080
Partnered ^b^	214	53.2	120	56.1	94	43.9	
**Sexual Orientation**							
Heterosexual/Straight	306	76.3	154	50.3	152	49.7	0.197
Others ^c^	95	23.7	55	57.9	40	42.1	
**Currently have a regular place of care**							
Yes	247	61.8	141	57.1	106	42.9	0.010
No	153	38.2	67	43.8	86	56.2	
**Currently have Health Insurance**							
Yes	378	94.5	202	53.4	178	46.6	0.026
No	22	5.5	6	27.3	16	72.7	
**Received healthcare provider recommendation**							
Yes	250	62.2	186	74.4	64	25.6	<0.001
No	152	37.8	23	15.1	129	84.9	

^a^ Dating/ Married/Living with a partner

^b^ Black/Asian/More than one race

^c^Gay/lesbian/bisexual/asexual/queer

*Column total may not add up to 402 due to missing values

### Unadjusted analysis

Table 2 shows the results of the unadjusted associations between predictor variables and HPV vaccination status. Variables associated with HPV vaccination in unadjusted analysis included sex, having a regular place of healthcare, having health insurance, healthcare provider recommendation, HPV knowledge, perceived risk, perceived severity, perceived benefits, perceived barrier, and self-efficacy. Variables not associated with HPV vaccination status included age, race, ethnicity, relationship status and sexual orientation.

**Table 2:** Bivariate association between predictor variables and HPV vaccination status.

Variables	Unadjusted OR (95% CI)	P value
**Age (years)**	1.06 (0.96, 1.17)	0.275
**Sex**		<0.001
Female	Ref	
Male	0.39 (0.24, 0.62)	
**Race**		0.255
White	Ref	
Others ^b^	0.69 (0.37, 1.31)	
**Ethnicity**		0.575
Non-Hispanic	Ref	
Hispanic	1.27 (0.55, 2.93)	
**Relationship Status**		0.081
Single	Ref	
Partnered ^a^	1.42 (0.96, 2.10)	
**Sexual Orientation**		0.2326
Heterosexual/Straight	Ref	
Others ^c^	1.32 (0.83, 2.1)	
**Currently have a regular place of healthcare**		0.010
Yes	1.71 (1.14, 2.56)	
No	Ref	
**Currently have Health Insurance**		0.022
Yes	3.06 (1.17, 7.99)	
No	Ref	
**Received healthcare provider recommendation**		<0.001
Yes	16.30 (9.63, 27.60)	
No	Ref	
**HPV Knowledge**	1.32 (1.20, 1.46)	<0.001
**Perceived Risk**	1.18 (1.12, 1.24)	<0.001
**Perceived Severity**	1.12 (1.03, 1.21)	0.006
**Perceived Benefit**	1.26 (1.18, 1.35)	<0.001
**Perceived Barrier**	0.69 (0.63, 0.75)	<0.001
**Self-efficacy**	1.38 (1.26, 1.51)	<0.001

CI: confidence interval, OR: odds ratio

^a^ Dating/ Married/Living with a partner

^b^ Black/Asian/More than one race

### Multivariable logistic regression model

Table 3 presents the adjusted odds ratios and 95% confidence intervals for the results of the final multivariable logistic regression. In the final adjusted model, the odds of being vaccinated were higher among individuals who received healthcare provider recommendations compared with those who did not (adjusted OR = 12.90; 95% CI: 7.08, 23.51). Additionally, those who had higher perceived risk (adjusted OR = 1.12; 95% CI: 1.05, 1.20) and higher HPV knowledge (adjusted OR = 1.17; 95% CI: 1.03, 1.33) had higher odds of being vaccinated. Lastly, the odds of being vaccinated were lower among participants with higher perceived barriers (adjusted OR = 0.73; 95% CI: 0.66, 0.81). The result of the Hosmer-Lemeshow test was not statistically significant indicating the model had a good fit to the data (χ^2^ = 8.02, p = 0.4317).

**Table 3:** Final Multivariable logistic regression model results showing significant variables associated with HPV vaccination.

	Adjusted OR (95% CI)	P value
Perceived Risk	1.12 (1.05, 1.20)	0.001
Perceived Barriers	0.73 (0.66, 0.81)	<0.001
HPV Knowledge	1.17 (1.03, 1.33)	0.015
Received healthcare provider recommendation		<0.001
Yes	12.90 (7.08, 23.51)	
No	Ref	

CI: confidence interval, OR: odds ratio

### Preferences for HPV vaccine information and promotion strategies

**Fig 1:**
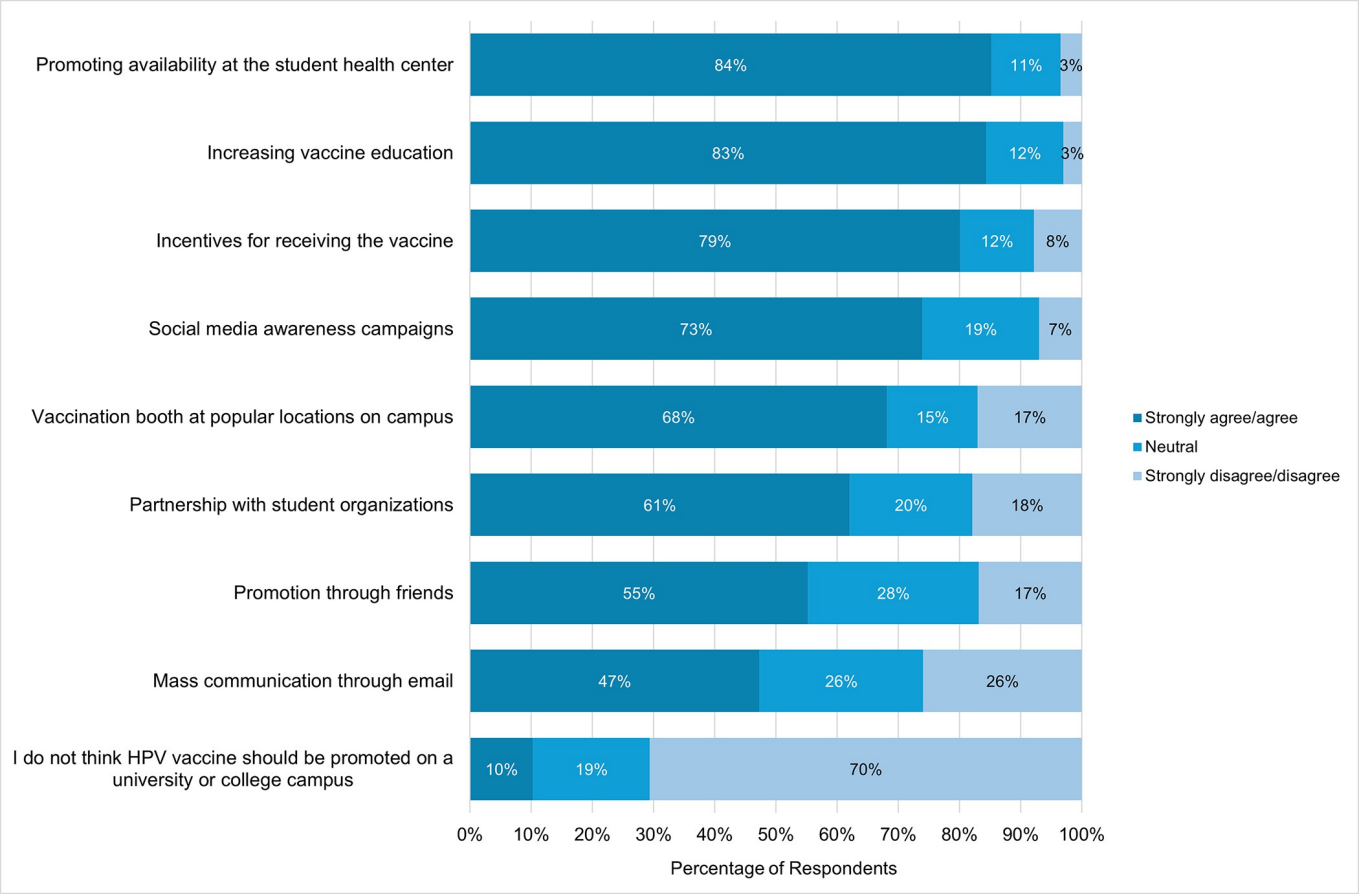
Preferred HPV vaccine promotion strategies.

Participants selected their level of agreement with various HPV vaccine promotion strategies. The frequency of participants who agreed/strongly agreed to the different promotion strategies included (61%), and promotion through friends (55%). About 10% of participants agreed that the HPV vaccine should not be promoted on a university campus (Fig 1).

**Fig 2:**
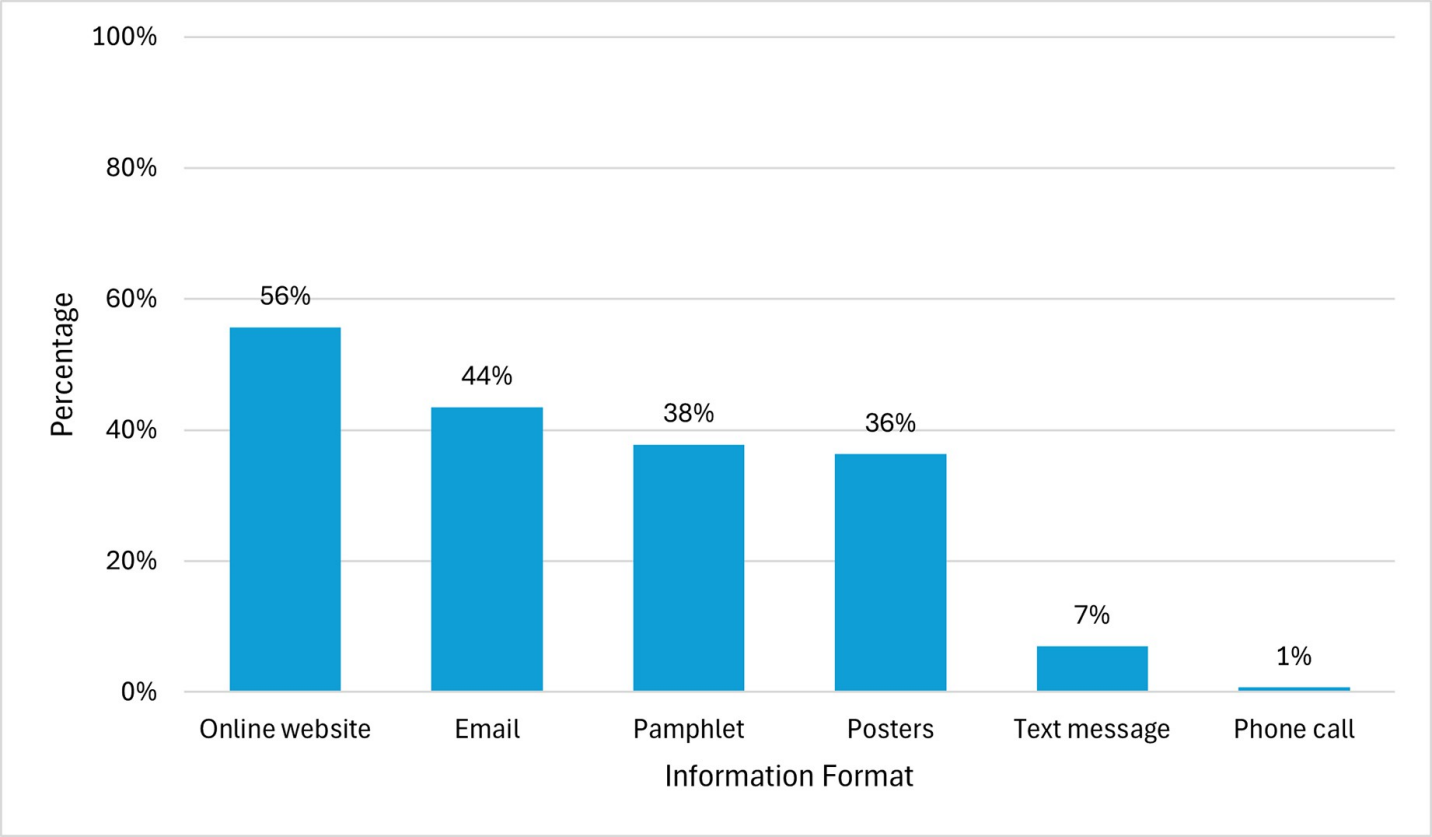
Preferred HPV vaccine information format.

**Fig 3:**
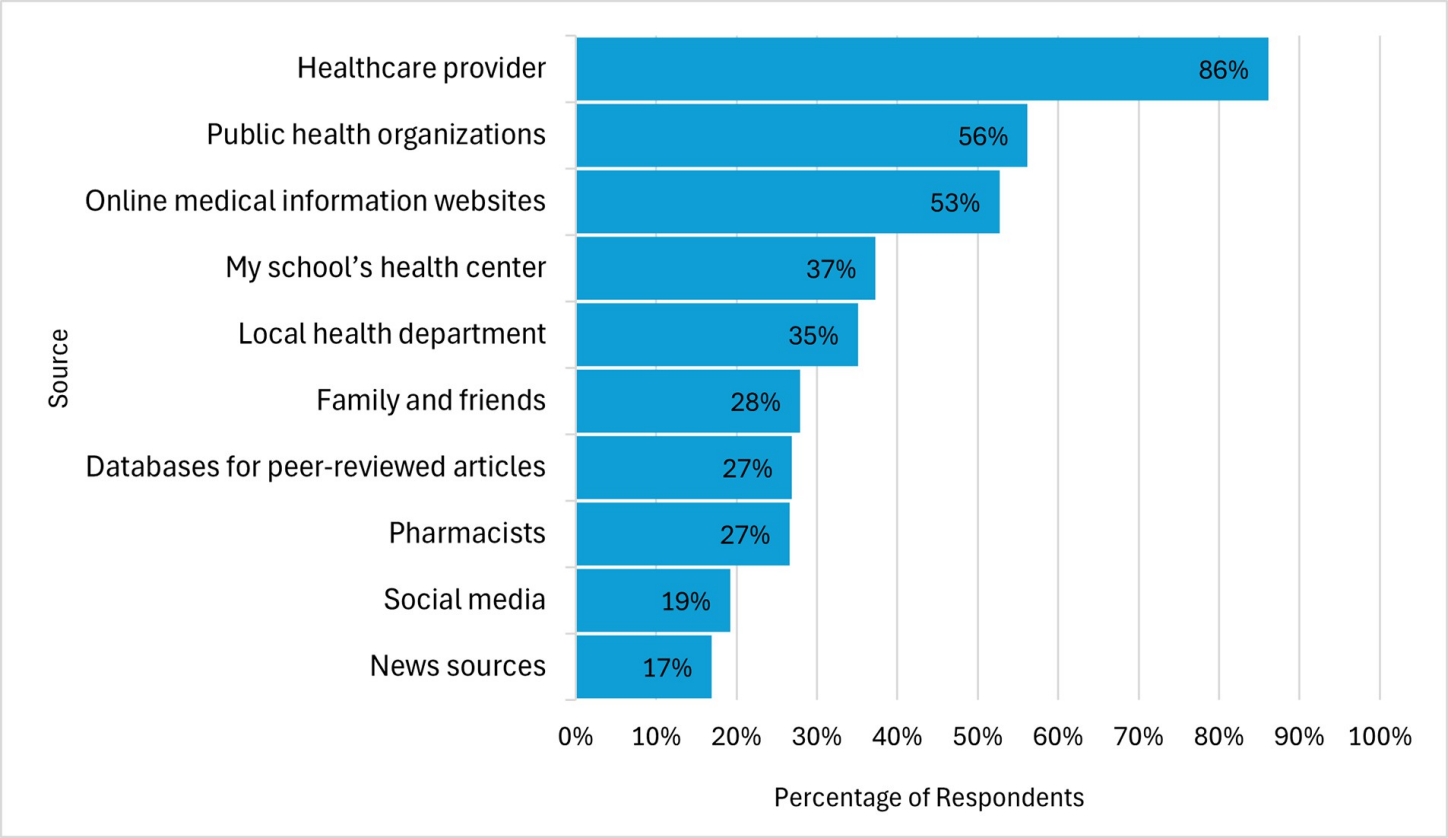
Preferred source of HPV vaccine information.

**Fig 4:**
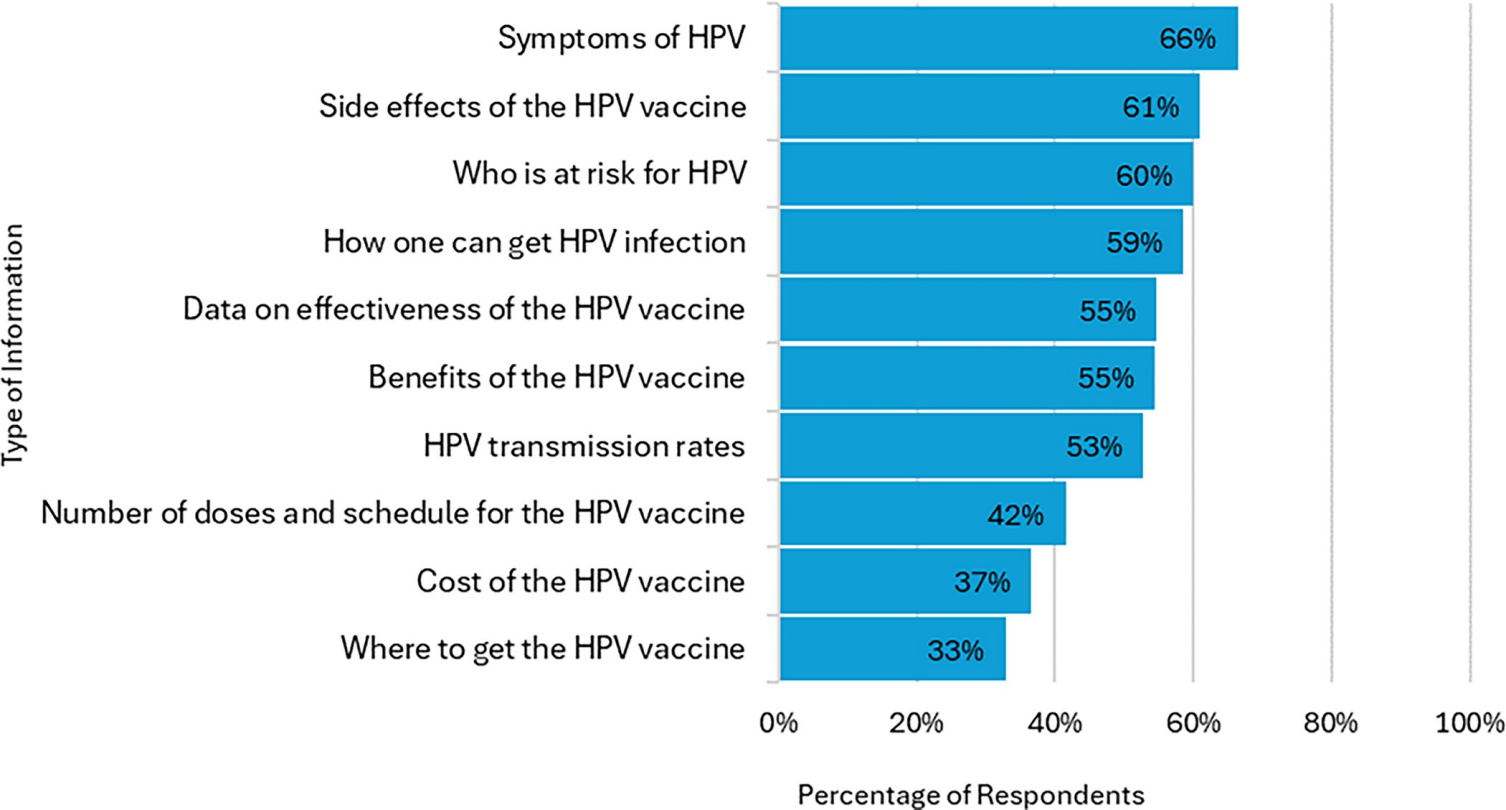
Preferred type of information.

In terms of preferred HPV vaccine information format, the two most commonly selected formats were online website (56%) and email (44%), while the least frequently selected were text message (7%) and phone call (0.7%) (Fig 2). The most frequently selected HPV vaccine sources of information were healthcare providers (86%), public health organizations (56%), and online medical information websites (53%). The least frequently reported sources of HPV vaccine information were news sources (17%) and social media (19%) (Fig 3). Participants selected different types of information that they would be interested in learning about HPV infection and the HPV vaccine. The frequencies of participants preferences for type of information are displayed in Fig 4.

## Discussion

This study focused on factors associated with HPV vaccination among young adults and HPV vaccine promotion and information preferences. Findings from this study showed that perceived risk, perceived barriers, HPV knowledge, and receipt of healthcare provider recommendations were associated with HPV vaccination.

One relevant finding from this study was that a lower proportion of males reported being vaccinated when compared with females. This finding corroborates reports from previous studies that males are less likely to receive the HPV vaccine [36], and vaccination rates have been historically low among males compared with females [37, 38]. Promoting HPV vaccination among both women and men is of utmost importance since oropharyngeal cancer is prevalent among men and is increasing, accounting for over 82% of newly diagnosed HPV-related cancer among men [8]. This highlights the need for both men and women to be vaccinated against HPV to prevent the development of these cancer types. Previous studies have shown that the low uptake of the HPV vaccine among males may be attributed to their inadequate knowledge and awareness about the vaccine [30, 39]. Additionally, the lower vaccine uptake among males may be attributed to the initial recommendation for females only when the HPV vaccine was first introduced in 2006. Future efforts to improve HPV vaccination among males may consider interventions to increase awareness and knowledge.

As expected, another finding from this study was that those who reported receiving healthcare provider recommendations had a higher likelihood of being vaccinated. Although the estimate suggesting the association of HPV vaccination with reporting receipt of healthcare provider recommendation is large, interpretation should be made with caution due to the wide distribution of the confidence interval. Nonetheless, the influential role of healthcare providers has been documented in previous literature as individuals who received a recommendation are more likely to initiate and complete the HPV vaccine series [40–42]. In one study among adults, the odds of HPV vaccine uptake was 18 times among those who received provider recommendations compared with those who did not [41]. Another study showed that about 70% of participants reported that their healthcare provider’s recommendation would be most influential in receiving the HPV vaccine [15]. Individuals may also report that they have not received HPV vaccine recommendations. In a previous study, about 53% of unvaccinated adults reported that lack of provider recommendation was a factor preventing them from getting vaccinated [43]. Other studies have reported similar values with about 51% of participants in one study among college students reporting lack of HPV vaccine healthcare provider recommendations [44]. For this study, about 38% reported not receiving healthcare provider recommendations about the HPV vaccine. It is unclear whether individuals with positive attitudes towards the HPV vaccine and willingness to accept the HPV vaccine may be more likely to report receiving recommendations from their healthcare providers. Given that evidence from literature suggests that healthcare provider recommendation is important for increasing HPV vaccination rates, future research considerations may involve exploring reasons for the lack of provider recommendation among adults.

Results from this study showed a significant association between HPV knowledge and HPV vaccination. Previous studies have shown mixed results regarding the relationship between knowledge and HPV vaccination with some studies finding that increased knowledge is associated with HPV vaccination [31, 44] while others do not [34, 45]. Notwithstanding, students have frequently reported inadequate knowledge or awareness as a barrier to vaccination. In the study by Oh and colleagues, 51% of students reported inadequate knowledge as a factor preventing them from being vaccinated [44]. This highlights the need for evidence-based HPV vaccine education and promotion to improve knowledge and awareness among young adults.

Perceived barrier was investigated in this study as an aggregate of individual factors that may prevent HPV vaccine uptake. Study findings suggests an association between perceived barriers and HPV vaccination. It is imperative to address individual barriers that may hinder the uptake of the HPV vaccine among young adults. One barrier that may hinder vaccine uptake is the cost associated with completing the HPV vaccine series. Although most private insurance and Medicaid plans are required to cover ACIP recommended vaccines with no patient costs [46], individuals who lack health insurance coverage may not be able to afford the out-of-pocket expenses associated with completing the three doses of the HPV vaccine series. Furthermore, individuals without regular access to health care due to lack of insurance may miss opportunities for preventive care, including immunizations, as previous studies suggest an association between more doctor’s visits and initiation and completion of HPV vaccination [36, 47]. This highlights the importance of increasing awareness regarding available health insurance coverage options for vaccination among individuals with insurance and no-cost options for individuals without insurance. Among individuals without regular health care, it is important to promote resources on alternative vaccination locations such as pharmacies and local health departments.

Individual concerns about whether the HPV vaccine is safe and effective is worth mentioning as a factor that may hinder acceptance of vaccination. Recent reports have shown that safety concerns about the HPV vaccine are frequent and have continued to increase over time in the United States [48]. Previous research also indicates that those who reported concerns about HPV vaccine safety as a barrier were less likely to be vaccinated [43]. Since rigorous evidence supports the safety and efficacy of the HPV vaccine [1], future efforts may consider exploring options to allay fears regarding the safety and side effects of the HPV vaccine among young adults.

Most participants in this study agreed that promoting the HPV vaccine on campus through their school’s student health center is a preferred vaccination promotion strategy. However, the implementation of programs to improve vaccination rates may differ across student health services. For example, a previous study conducted among student health centers found that institutions less frequently implement strategies to improve HPV vaccine uptake compared to influenza, Tdap (Tetanus, Diphtheria, Pertussis), and MMR (Measles, Mumps, Rubella) vaccines [49]. Furthermore, only about half of the student health centers that participated in the same study stocked and administered the HPV vaccine [49]. Several factors may influence the ability of a student health center to provide the HPV vaccine, including cost or financial reasons, lack of vaccine mandate or school requirements, or prioritization of other vaccines [49]. Notwithstanding, student health centers that do not have the capacity to offer the HPV vaccine could implement different strategies, including increasing education and campaigns on campus, routine counseling, and referral of students to alternative vaccination locations such as health departments and pharmacies [49]. For student health centers that provide the HPV vaccine, additional efforts may be needed to publicize vaccine availability or increase HPV vaccine assessment and recommendation by providers since students have previously reported lack of knowledge that the HPV vaccine is available at their institution’s student health center [31].

Another HPV vaccination promotion strategy commonly selected by participants in this study is social media awareness campaigns. This finding is consistent with previous studies where college students have reported digital or social media as a preferred way to propagate HPV vaccine information [25, 50]. Given that social media campaign is favored by young adults and provides an opportunity to promote HPV vaccination, it is crucial to address inaccurate information or misinformation on social media platforms that may deter vaccine uptake [50, 51]. This study also describes HPV vaccination information preferences, which could be useful for targeted educational programs aimed at improving vaccination rates among young adults. For the type of HPV vaccine information, more than half of study participants indicated that they would be interested in learning about HPV symptoms, side effects of HPV vaccine, who is at risk for HPV, ways individuals can get HPV, effectiveness data, vaccine benefits, and HPV transmission rates. Additionally, most respondents in this study indicated a preference for online websites as their preferred communication formats. It is important to consider incorporating these HPV information needs and formats into future educational outreach to HPV vaccine catch-up population.

This study has some limitations. First, this study utilized data from a convenience sample of young adults on a single university campus, hence, findings may not apply to young adults outside of the college environment or even to other college populations. The generalizability of the study findings may be limited. Second, the study survey was self-reported which may introduce biases that limits that validity of study findings. Third, participants’ responses may be subject to social-desirability bias given that HPV is transmitted sexually. However, the online survey was anonymous, and participants were informed that their responses were confidential. Despite study limitations, this study provides valuable insights into factors related to HPV vaccination among young adults in Tennessee. Findings from this study regarding preferences for HPV vaccine information will be useful for developing targeted HPV vaccine informational materials.

### Conclusion

HPV vaccination remains suboptimal among young adults. Future HPV vaccination efforts may consider focusing on interventions or programs among both college males and females. Additionally, future research efforts may consider exploring interventions involving healthcare providers that serve university or college student populations through student health services. HPV vaccine educational programs may consider incorporating preferred information and promotion strategies among young adults.

## Supporting information

S1 File (DOCX)
