# Supplementary material for: Examining HPV vaccination behavior among young adults: Insights from applying the Health Belief Model
Source: PLoS One. 2024 Nov 1;19(11):e0312700. doi: 10.1371/journal.pone.0312700 (PMC11530009; doi:10.1371/journal.pone.0312700)
Supplement: S1 File — (DOCX) [file pone.0312700.s001.docx]

**Manuscript Title: Examining HPV Vaccination Behavior Among Young Adults: Insights from applying the Health Belief Model**

| **Measures** | **Survey Items** |
| --- | --- |
| Perceived Risk | Please rate your level of agreement with each statement (5 point Likert scale response from Strongly Agree to Strongly Disagree)   - I consider myself at risk for HPV infection - If I don't get vaccinated for HPV, I would feel at risk for getting HPV infection sometime in the future - If I don't get vaccinated for HPV, I would feel at risk for getting HPV-related cancer sometime in the future - If I don’t get vaccinated for HPV, I am likely going to get infected with HPV in the future - If I don’t get vaccinated for HPV, I am likely going to develop HPV related cancers in the future |
| Perceived Severity | Please rate your level of agreement with each statement (5 point Likert scale response from Strongly Agree to Strongly Disagree)   - Being infected with HPV would be very serious for me - I worry about the negative impact of being diagnosed with HPV infection - Being diagnosed with HPV-related cancer (anal, oral, or penile) would have negative impact on my life *(for males only)* - Being diagnosed with HPV-related cancer (anal, oral, or penile) would be devastating for me *(for males only)* - Being diagnosed with HPV-related cancer (cervical, anal, oral) would have negative impact on my life *(for females only)* - Being diagnosed with HPV-related cancer (cervical, anal, oral) would be devastating for me *(for females only)* |
| Perceived Benefit | Please rate your level of agreement with each statement (5 point Likert scale response from Strongly Agree to Strongly Disagree)   - I think the HPV vaccine will be beneficial to me - If I get vaccinated for HPV, I am protecting myself from future risk of HPV-related cancers including cervical, anal, and oral cancers *(for females only)* - If I get vaccinated for HPV, I am protecting myself from future risk of HPV-related cancers including anal, oral, and penile cancers *(for males only)* - If I get vaccinated for HPV, I am protecting my current or future sexual partner(s) from HPV infection - Getting vaccinated for HPV will reduce my chances of getting infection - Getting vaccinated for HPV will reduce my chances of getting HPV related cancer |
| Perceived Barriers | How much would the following factors prevent you from getting vaccinated for HPV? (5 point Likert scale response from Strongly Agree to Strongly Disagree)   - Concerns about the possible side effects of the HPV vaccine - If the vaccine cost is too much - Concerns about whether the vaccine is safe |
| Self-efficacy | Please rate your level of agreement with each statement (5 point Likert scale response from Strongly Agree to Strongly Disagree)  *For unvaccinated*   - I have the information I need to decide on getting the HPV vaccine - I feel confident in my ability to get vaccinated for HPV - I feel confident in my ability to get vaccinated for HPV, even if it means finding the time to go to the doctor three times within 12 months   *For vaccinated*   - I had the information I needed when I decided to get the HPV vaccine - I felt confident in my ability to get vaccinated for HPV - I felt confident in my ability to get vaccinated for HPV, even if it meant finding the time to go to the doctor three times within 12 months |
| Cues to action: Role of Healthcare Providers | Have you received recommendations from your healthcare provider about the HPV vaccine? (Yes/No) |
| HPV Knowledge | Please select an option to the following questions (yes, no, do not know):   - HPV is the most common sexually transmitted disease - HPV can cause cervical cancer - HPV can cause cancer of the back of the throat - HPV can cause anal cancer - Most people with genital HPV have no visible signs or symptoms - Vaccination will prevent certain types of HPV |
| Preferred HPV Vaccine Promotion Strategies | Which of the following promotion strategies on a university or college campus would you consider useful for increasing HPV vaccination? (5 point Likert scale response from Strongly Agree to Strongly Disagree)   - Vaccination booth at popular locations on campus - Social media awareness campaigns - Incentives for receiving the vaccine - Mass communication through email - Partnership with student organizations such as sororities and fraternities - Promoting availability at the student health center - Increasing vaccine education - Promotion through friends - I do not think HPV vaccine should be promoted on a university or college campus |
| Preferred HPV Vaccine Information Format | In which of the following formats would you like to learn about HPV vaccine? (select all that apply)   - Pamphlet - Posters - Email - Online website - Text message - Phone call |
| Preferred Source of HPV Vaccine Information | Where would you go to find information about the HPV vaccine? (Select all that apply)   - Healthcare provider - Public health organization, e.g., Centers for Disease Control and Prevention (CDC) - Local health department - Family and friends - News sources, e.g., television - Pharmacists - Online medical information websites e.g. Mayo Clinic - Social media - My school’s health center - Databases for peer-reviewed articles - Other (please specify) |
| Preferred Type of Information | What type of information would you be interested in learning about HPV infection and the HPV vaccine? (select all that apply)   - Symptoms of HPV - Who is at risk for HPV - How one can get HPV infection - HPV transmission rates - Benefits of the HPV vaccine - Side effects of the HPV vaccine - Number of doses and schedule for the HPV vaccine - Where to get the HPV vaccine - Data on effectiveness of the HPV vaccine - Cost of the HPV vaccine |

| **Summary Statistics and Internal Reliability for HBM Constructs** | | | | | |
| --- | --- | --- | --- | --- | --- |
| **Scale** | **Mean Score** | **SD** | **Minimum score** | **Maximum Score** | **Cronbach’s alpha** |
| Perceived susceptibility | 13.96 | 4.2 | 5 | 25 | 0.853 |
| Perceived severity | 17.0 | 2.5 | 8 | 20 | 0.752 |
| Perceived benefits | 20.9 | 3.6 | 5 | 25 | 0.934 |
| Perceived barriers | 8.8 | 2.9 | 3 | 15 | 0.708 |
| Self-efficacy | 10.8 | 2.7 | 3 | 15 | 0.803 |
| SD: Standard Deviation | | | | | |

**References**

1. Ratanasiripong, N.T., A.L. Cheng, and M. Enriquez, *What college women know, think, and do about human papillomavirus (HPV) and HPV vaccine.* Vaccine, 2013. **31**(10): p. 1370-6.

2. Kellogg, C., et al., *A significant portion of college students are not aware of HPV disease and HPV vaccine recommendations.* Human Vaccines & Immunotherapeutics, 2019. **15**(7-8): p. 1760-1766.

3. Gerend, M.A. and J.E. Shepherd, *Predicting Human Papillomavirus Vaccine Uptake in Young Adult Women: Comparing the Health Belief Model and Theory of Planned Behavior.* Annals of Behavioral Medicine, 2012. **44**(2): p. 171-180.

4. Johnson, C. and R. Ogletree, *Knowledge and Behavioral Intention Related to HPV Vaccination Among Male College Students.* American Journal of Health Education, 2017. **48**(5): p. 320-330.
